# Semi-supervised segmentation of metastasis lesions in bone scan images

**DOI:** 10.3389/fmolb.2022.956720

**Published:** 2022-10-28

**Authors:** Qiang Lin, Runxia Gao, Mingyang Luo, Haijun Wang, Yongchun Cao, Zhengxing Man, Rong Wang

**Affiliations:** ^1^ School of Mathematics and Computer Science, Northwest Minzu University, Lanzhou, China; ^2^ Key Laboratory of China’s Ethnic Languages and Information Technology of Ministry of Education, Northwest Minzu University, Lanzhou, China; ^3^ Key Laboratory of Streaming Data Computing Technologies and Application, Northwest Minzu University, Lanzhou, China; ^4^ Department of Nuclear Medicine, Gansu Provincial Hospital, Lanzhou, China

**Keywords:** bone scan, metastasis, lesion segmentation, deep learning, semi-supervised learning

## Abstract

To develop a deep image segmentation model that automatically identifies and delineates lesions of skeletal metastasis in bone scan images, facilitating clinical diagnosis of lung cancer–caused bone metastasis by nuclear medicine physicians. A semi-supervised segmentation model is proposed, comprising the feature extraction subtask and pixel classification subtask. During the feature extraction stage, cascaded layers which include the dilated residual convolution, inception connection, and feature aggregation learn the hierarchal representations of low-resolution bone scan images. During the pixel classification stage, each pixel is first classified into categories in a semi-supervised manner, and the boundary of pixels belonging to an individual lesion is then delineated using a closed curve. Experimental evaluation conducted on 2,280 augmented samples (112 original images) demonstrates that the proposed model performs well for automated segmentation of metastasis lesions, with a score of 0.692 for DSC if the model is trained using 37% of the labeled samples. The self-defined semi-supervised segmentation model can be utilized as an automated clinical tool to detect and delineate metastasis lesions in bone scan images, using only a few manually labeled image samples. Nuclear medicine physicians need only attend to those segmented lesions while ignoring the background when they diagnose bone metastasis using low-resolution images. More images of patients from multiple centers are typically needed to further improve the scalability and performance of the model *via* mitigating the impacts of variability in size, shape, and intensity of bone metastasis lesions.

## 1 Introduction

A bone scan (skeletal scintigraphy) with technetium-99 methylenediphosphonic acid (^99m^Tc-MDP) is one of the most commonly used clinical tools for screening bone metastasis (Sderlund, 1996; Costelloe et al., 2009). When a primary solid tumor invades into the bone tissue, there will be one or more areas of increased radionuclide uptake in a ^99m^Tc-MDP single-photon emission computed tomography (^99m^Tc-MDP SPECT) image. Compared to positron emission tomography (PET) imaging, a ^99m^Tc-MDP SPECT bone scan is more available and affordable for surveying skeletal metastases due to its high sensitivity and low cost (Moon et al., 1998).

However, ^99m^Tc-MDP SPECT imaging suffers from low specificity typically caused by inferior spatial resolution, accumulation of radiopharmaceuticals in the normal bones, soft tissues or viscera, and uptake in benign processes (Nathan et al., 2013). Low specificity combined with the normal variation of uptake and technical artifacts often brings misinterpretation to human experts when they manually diagnose bone metastasis.

Automated analysis of ^99m^Tc-MDP SPECT images is desired for accurate and efficient diagnosis of bone metastasis. Conventional machine learning algorithms have been adopted to develop methods for identifying bone metastasis (Aslanta et al., 2016; Elfarra et al., 2019; Mac et al., 2021; Sadik et al., 2006; Sadik et al., 2008) or delineating metastasis lesions (Cheimariotis et al., 2018; Thorwarth et al., 2013; Zhu et al., 2008). However, the handcrafted image features often have insufficient capability and unsatisfied performance for clinical tasks (Shan et al., 2020).

Deep learning has been revolutionizing the field of machine learning for the past decades. As a mainstream branch of deep learning, convolutional neural networks (CNNs) have gained huge success in computer vision due to their superiority in automatically learning hierarchical features from images in an optimal way. Several excellent review articles present a holistic perspective on the recent progress of deep learning in medical image segmentation (AsgariTaghanaki et al., 2021; Minaee et al., 2022; Litjens et al., 2017; Lei et al., 2020). Semi-supervised learning is becoming one of the hot research topics in this field due to the reduced requirement of large-scale labeled images (Christoph et al., 2017; Doulamis and Doulamis, 2014; Tarvainen and Valpola, 2017).

There has recently been a substantial amount of CNN-based work aimed at developing image classification methods for automated detection or diagnosis of metastasis (Bochkovskiy et al., 2020; Cheng et al., 2021a; Cheng et al., 2021b; Dang, 2016; Guo et al., 2022; Lin et al., 2021a; Lin et al., 2021b; Lin et al., 2021c; Li et al., 2022; Papandrianos et al., 2020a; Papandrianos et al., 2020b; Papandrianos et al., 2020c; Pi et al., 2020; Redmon and Farhadi, 2018; Zhao et al., 2020) by classifying ^99m^Tc-MDP SPECT images into categories. By contrast, segmenting ^99m^Tc-MDP SPECT images to detect and delineate metastasis lesions is still in its infancy. Using the recurrent CNN (RCNN) (Liang and Hu, 2015) as a backbone network, Chen and Frey (2020) proposed a semi-supervised segmentation model to delineate bone structures in the pelvis. Their model reported a score of 0.593 for the Dice metric on the simulated instead of real clinical SPECT images. MaligNet (Apiparakoon et al., 2020) is a two-stage network used for semi-supervised segmentation of chest metastasis lesions, consisting of a feature extraction subnetwork (ResNet-50) and feature classification subnetwork (ladder feature pyramid network). Their proposed network achieved a mean score of 0.848 for the F-1 score, without segmentation metrics [e.g., Dice and IoU (intersection over union)] being reported. Based on the classical networks U-Net (Ronneberger et al., 2015) and R-CNN (He et al., 2020), we investigated supervised segmentation of bone metastasis lesions in clinical ^99m^Tc-MDP SPECT images (Lin et al., 2020), obtaining a best score of 0.6103 for the IoU metric.

Using clinical ^99m^Tc-MDP SPECT bone scans, we have propose an RCNN-based method for segmenting bone metastasis lesions in a semi-supervised way in this work. The proposed segmentation method can automatically identify a lesion and delineate its boundary using only few manually labeled samples (i.e., semi-supervised learning). Our work is based on the following observations.

First, image segmentation involving partitioning images into multiple segments or objects (e.g., organs, tissues, and lesions) is routinely conducted in clinical diagnosis. This thereby enables the extraction of bone metastatic lesions and measurement of lesion volumes. Second, 2D bone scan is characterized by inferior spatial resolution. The size of a whole-body image is 256 (width) × 1024 (height). This brings a huge challenge to manual analysis by nuclear medicine physicians. Lastly, the lack of large labeled data sets of bone scan images is an especially prevalent challenge in supervised image segmentation because it is very time-consuming, laborious, and subjective to manually labeling lesions in large-sized low-resolution ^99m^Tc-MDP SPECT images. On the contrary, CNN-based semi-supervised segmentation has the potential to automatically divide an image into regions of concerns with only a small number of partially labeled samples.

Given that the spine and ribs are the common areas where primary tumors frequently invade , a whole-body SPECT image was first cropped to extract the thoracic region in this work. With the extracted regional images, an end-to-end semi-supervised segmentation network was built to learn the hierarchal representations of ^99m^Tc-MDP SPECT images and classify all pixels into categories (i.e., the lesion and background). The pixels falling into each lesion were then surrounded by an irregular closed curve. The delineated lesions could help the human expert focus on bone metastasis lesions while ignoring the background area, thereby enabling to improve the accuracy and efficiency of the diagnosis.

The main contributions of this work are first, we define the research problem of segmenting metastasis lesions in ^99m^Tc-MDP SPECT images in a semi-supervised way. Second, we propose a semi-supervised segmentation method consisting of image feature extraction and pixel classification. Last, we utilize a set of clinical ^99m^Tc-MDP SPECT images to evaluate the proposed method. The experimental results show that our method performs well in the automated segmentation of bone metastasis lesions with only few manually labeled samples being used.

The remaining part of this article is organized as follows: the proposed semi-supervised segmentation method is detailed in Section 2. Experimental evaluation conducted on clinical data is provided in Section 3. A brief discussion is presented in Section 4. We have concluded this work and pointed out the future research directions in Section 5.

## 2 Methodology

**FIGURE 1:**
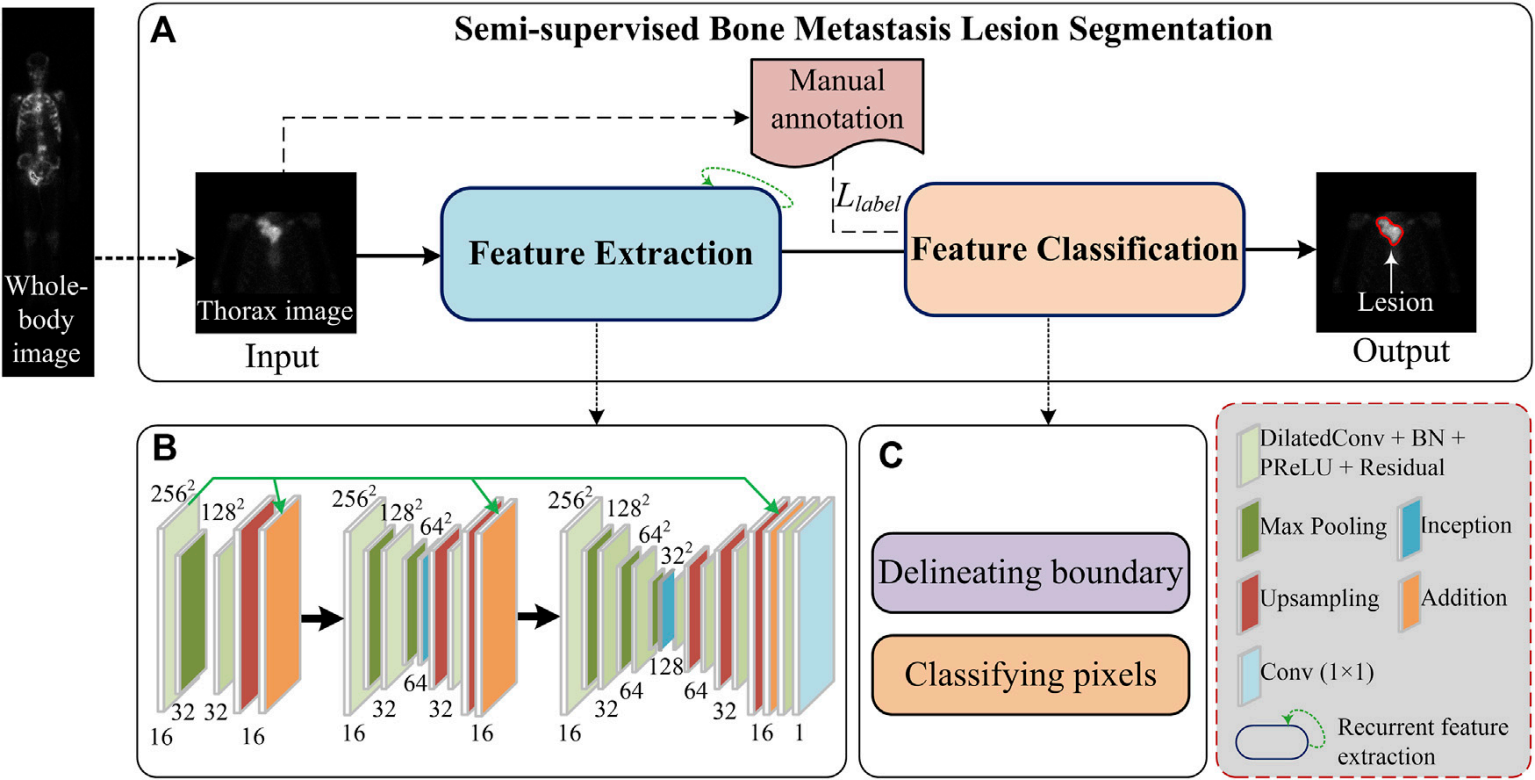
Overview of the proposed semi-supervised metastasis lesion segmentation method.

An overview of the proposed semi-supervised segmentation framework is depicted in Figure 1A, comprising two stages, namely, image feature extraction (Figure 1B) and pixel classification (Figure 1C). During the feature extraction stage, a set of cascaded layers is used to learn low-to-high representations of low-resolution images, enabling us to extract features of bone metastasis lesions as much as possible. During the pixel classification stage, the pixels within an image are classified into categories with the partially labeled samples (semi-supervised) and the boundary of pixels belonging to a lesion is delineated.

### 2.1 Image cropping

Whole-body SPECT bone scanning outputs large images with a size of 256 pixels × 1024 pixels. This brings a heavy computational burden to the pixel-level classification task. On the other hand, the thoracic region that covers the sternum, clavicles, scapulae, and ribs is the most common site of metastases in a variety of solid cancers (Nathan et al., 2013). Focusing on the automated segmentation of bone metastasis lesions in the thoracic skeleton, an empirical image-cropping method (Li et al., 2022) is used to extract the thoracic region from the whole-body images. The cropping method contains three main steps: “whole-body image → body area,” “body area → upper body,” and “upper body → thoracic region.” A cropped regional image has the size of 256 × 256. Each “pixel” in this image is a 16-bit unsigned integer, representing the detected count of the radiotracer’s uptake.

A regional image can be viewed as a count matrix **CM**, which can be formally represented as

$$\begin{array}{l} CM=\left(c_{i,j}\right)|1\leq i,j\leq 256\\ c_{i,j}\in \left[0,c_{\max}\right]. \end{array}$$ (1)

Typically, *c* = 0 denotes the background pixels, and *c*_max_ varies largely from patient to patient. As mentioned previously, apart from the metastasized bone, the high uptake of radiopharmaceuticals is commonly seen in normal bones and benign processes. It is thus difficult to normalize the count, *c*, into a fixed range, such as it is done in natural image analysis.

### 2.2 Lesion labeling

Semi-supervised learning tasks still need human manual labels to train a segmentation model, where the labeled lesions act as ground truth in the experiments. To help human experts (a chief physician aged 45 years, an associate chief physician aged 40 years, and a resident physician aged 33 years) to manually label a low-resolution SPECT image, we developed an annotation system based on the open-source online tool LabelMe (http://labelme.csail.mit.edu/Release3.0/).

Using the LabelMe-based annotation system, three experienced nuclear medicine physicians in our group independently labeled each image. Let *l* = <*p*_1_, *p*_2_, …, *p*_*m*_ = *p*_1_> denote a manual label, which is a closed curve consisting of points. For a bone metastasis lesion, if the difference between the areas surrounded by any two closed curves is not larger than the threshold *t*_*ΔA*_, we randomly select any one from the three labels as the ground truth; a new annotation process will start otherwise. Specifically, the area difference *ΔA* is defined using the Intersection over Union (IoU ) in Eq. 2.

$$\Delta A=1-\frac{A_{l1}\cap A_{l2}}{A_{l1}\cup A_{l2}},$$ (2)

where *A*_*lk*_ (*k* = 1, 2) represents the area of the closed curve *lk*. We assign a value of 5% for *t*_*ΔA*_ in the experiments.

During the supervised training stage, the manual annotation *L*_*label*_ in Figure 1A will be fed into the segmentation model.

### 2.3 Feature extraction

As depicted in Figure 1B, cascaded layers are used in the feature extraction stage which include the residual dilated convolution, pooling, feature aggregation, inception connection, upsampling, and traditional convolution to extract multi-scale features of lesions from low-resolution images.

#### 2.3.1 Dilated residual convolution

Bone metastasis lesions typically demonstrate variability in size, shape, and intensity. Extracting hierarchical features of lesions from low-resolution images is significantly challenging. Compared to the conventional convolution, a dilated convolution has the potential to systematically aggregate multi-scale contextual information without losing resolution (Yu and Koltun, 2015). On the other hand, the residual connection can alleviate the gradient vanishing and explosion problems.

**FIGURE 2:**
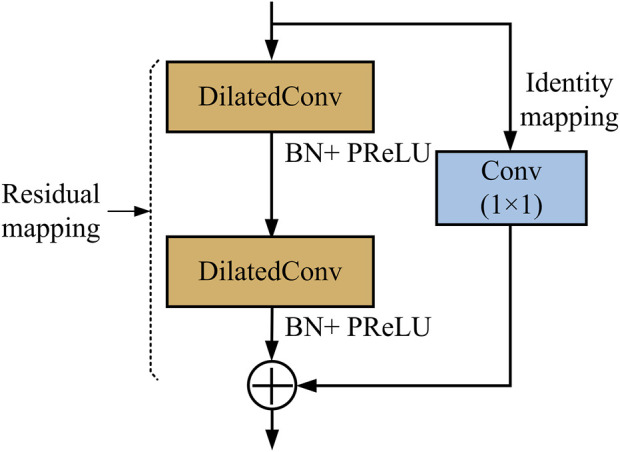
Structure of the residual dilated convolution block.

We used the residual dilated convolution block in the feature extraction stage to extract the multi-scale features of metastasis lesions, which is illustrated in Figure 2.

As shown in Figure 2, there are two paths in the residual dilated convolution block. In the residual mapping path, two dilated convolution (DilatedConv) layers are used to extract multi-scale features, with each followed by a batch normalization (BN) operation and parametric rectified linear unit (PReLU) operation. The BN operation has the potential to make a network utilize much higher learning rates and be less careful about initialization, enabling the acceleration of network training. As a typical activation function, PReLU performs a threshold operation to bring nonlinearity into the network. In the identity path (also skip path), a 1 × 1 conventional convolution is used to reduce the number of depth channels by simply mapping an input pixel to output pixel.

Given the *i* × *i* feature map *I*_*IN*_ with a kernel size of *k* as the input, for any given dilation rate of *d*, the size *o* of the feature map *I*_*OUT*_ after dilated convolution can be calculated according to Eq. 3.

$$\begin{array}{l} o=\left[\frac{i-2p-n}{s}\right]+1\\ n=k+\left(k-1\right)\left(d-1\right) \end{array},$$ (3)

where *p* and *s* denote the padding and stride, respectively.

#### 2.3.2 Inception architecture

Increasing the size of a network (depth and width) is one of the alternatives to improve the performance of a convolutional neural network. However, an enlarged network with a larger size is often prone to overfitting. The inception architecture (Szegedy et al., 2014) can find an optimal local sparse structure in the deep network while allowing for significantly increasing the number of units at each stage without an uncontrolled blow-up in computational complexity.

**FIGURE 3:**
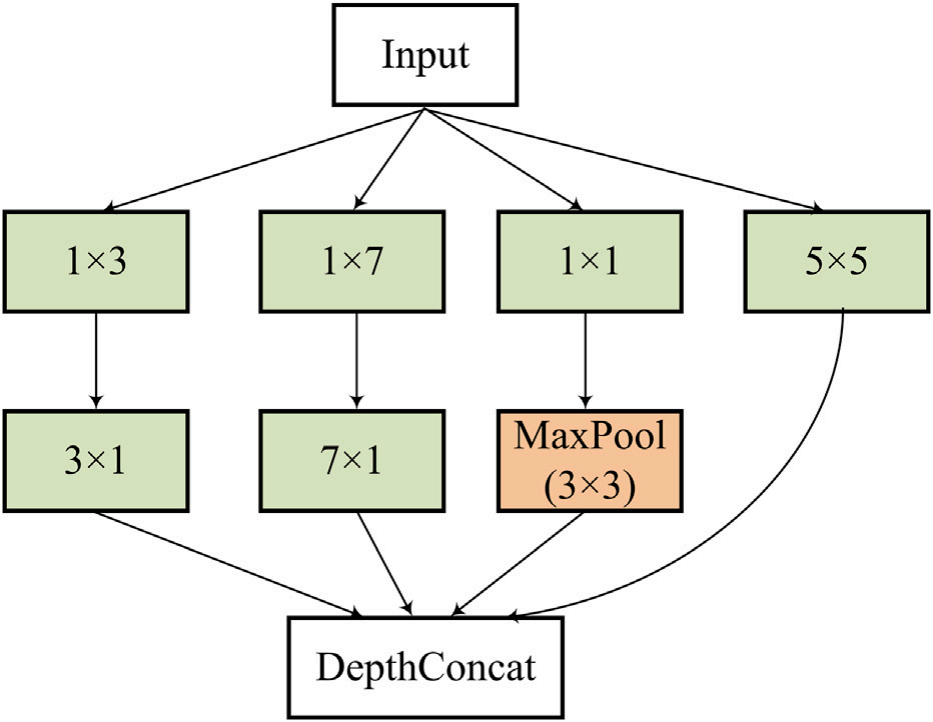
Structure of the inception block used in the feature extraction stage.

We define an inception block (see Figure 3) in this work to further extract multi-scale features of bone metastasis lesions.

The defined the inception block consists of several convolutions with different kernels (1 × 3/3 × 1, 1 × 7/7 × 1, 1 × 1, and 5 × 5) and a max pooling with stride = 2 after a 1 × 1 convolution to capture hierarchal features and halve the resolution of the grid. The DepthConcat concatenates the outputs from the previous layers.

#### 2.3.3 Feature aggregation

The dual views of 2D SPECT imaging are exploited to enhance the metastasis lesions by aggregating the anterior and posterior views of an image. Specifically, let *I*_*Ant*_ and *I*_*Post*_ be the anterior- and posterior-view images (features) of a patient, respectively, and an aggregated image *I*_*Agg*_ can be calculated according to Eq. 4.

$$I_{Agg}=f\left[I_{Ant},Mirr\left(I_{Post}\right)\right],$$ (4)

where *f* is the pixel-wise addition, and *Mirr* (∙) is the image horizontal flipping/mirroring operation.

**FIGURE 4:**
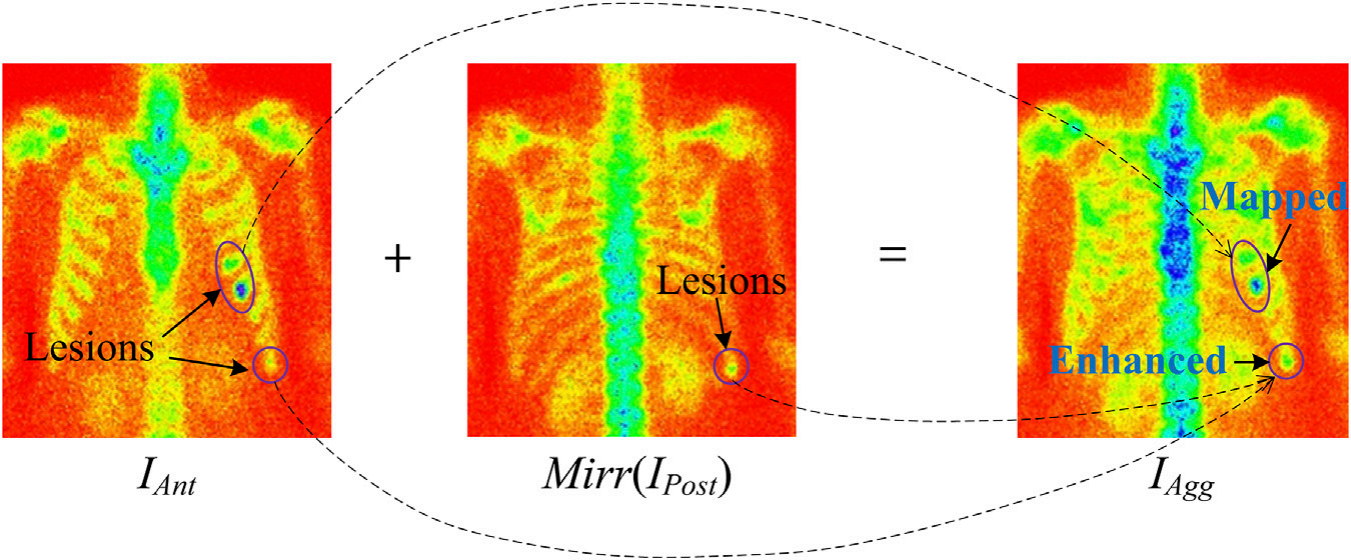
Illustration of feature aggregation using pixel-wise addition operation.

For instance, Figure 4 illustrates the image aggregation by using a pixel-wise addition operation (see “Addition” in Figure 1). Lesions in the aggregated image are either enhanced by adding the lesion areas in both the anterior and posterior views or mapping from the anterior or posterior views. For a patient with diagnosed bone metastasis, the lesion(s) can always be displayed in the aggregated image rather than only in the anterior or posterior one. The dual-view aggregation will be helpful for CNN-based classifiers in detecting lesions.

#### 2.3.4 Recurrent feature extraction

**FIGURE 5:**
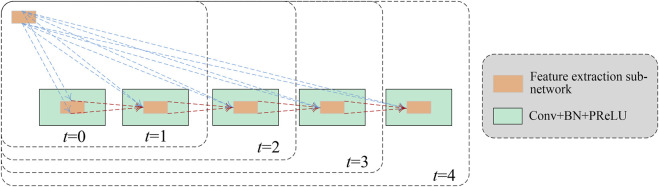
Structure of the recurrent feature extraction network with *t* indicating recurrence time.

Inspired by the structure of the recurrent convolutional layer in RCNN (Liang and Hu, 2015), we let the feature extraction network run in a recurrent way, indicated by the green dotted line with an arrow in Figure 1A, to integrate the context information for lesion segmentation. An illustration of a recurrent feature extraction subnetwork is depicted in Figure 5.

As shown in Figure 5, the feature extraction subnetwork comprises the feedforward network and recurrent network. Let *u*(*t*) be the input of the feedforward network at time *t*, and *x*(*t*–1) be the input of the recurrent network at time *t*–1, the output of the network at time *t* can be calculated according to Eq. 5.

$$z_{ijk}\left(t\right)={\left[W_{F\left(k\right)}\right]}^{T}u^{\left(i,j\right)}\left(t\right)+{\left[W_{R\left(k\right)}\right]}^{T}x^{\left(i,j\right)}\left(t-1\right)+b_{k}$$ (5)

where (*i*, *j*) indicates the pixel in the *k*-th feature map, *W*_*F*(*k*)_ and *W*_*R*(*k*)_ are the convolutional parameters of the feedforward and recurrent nets, and *b*_*k*_ is the bias.

### 2.4 Feature classification

Typically, image segmentation performs portioning of objects by automatically classifying pixels into the corresponding categories. In the paradigm of supervised learning, the labeled samples of images are used to train a segmentation model, which is then used to predict the class, *y*, from a pixel, *x*, of any new image. On the contrary, an unsupervised model is trained without manual labels.

Our semi-supervised segmentation model is trained using a large number of unlabeled samples together with only few labeled samples to build a pixel-level feature classifier. A core consideration of the semi-supervised segmentation model is in how to determine a segmentation loss function, which would probabilistically predict the class for each pixel and be defined according to the unsupervised segmentation loss and manual labels. The semi-supervised segmentation loss *ℓ* consists of two parts: unsupervised loss *ℓ*_*U*_ and supervised loss *ℓ*_*S*_, which is defined in Eq. 6.

$$\ell =\ell_{U}+\alpha \cdot \ell_{S}.$$ (6)

For a given image ***g***, let *c* = *f* (***g***) be a closed curve that delineates a bone metastasis lesion, the unsupervised loss *ℓ*_*U*_ in Eq. 6 can be defined as

$$\ell_{U}=v\cdot Area\left(f\left(g\right)>0\right)+\underset{f\left(g\right)>0}{\sum }{\left|g\left(x,y\right)-c_{1}\right|}^{2}+\underset{f\left(g\right)>0}{\sum }{\left|g\left(x,y\right)-c_{2}\right|}^{2}.$$ (7)

where *c*_1_ and *c*_2_ are the average of image ***g*** inside curve *c* and outside curve *c*, respectively; and *Area* (*∙*) is the function used for measuring the area inside the curve, which has been defined by Chan and Vese (2001).

Let *Ω* be the image filed and *u* be the label, the supervised segmentation loss *ℓ*_*S*_ can be calculated according to Eq. 8.

$$\ell_{s}=\sum_{f\left(i\right)}\left|\nabla \left(f\left(g\right)\right)\right|+\sum_{\Omega }\left({\left(1-\mu \right)}^{2}-{\left(0-\mu \right)}^{2}\right)f\left(g\right).$$ (8)

In the aforementioned Eqs. 7, 8, the constants are assigned as *α* = 0.4, *v* = 0.004, and *u* = 0 in the experiments.

With the semi-supervised segmentation loss function defined in Eq. 6, the segmentation model classifies each pixel into one of the categories (i.e., the background and lesion regions). The boundary of pixels that falls into an individual lesion is then delineated using a closed curve. For an input image, the segmented lesions act as the output of a segmentation model.

## 3 Results

### 3.1 Experimental data

The SPECT images used in this retrospective study were acquired from the Department of Nuclear Medicine, Gansu Provincial Hospital. A total of 724 whole-body images were collected from 362 patients, who were clinically diagnosed with bone metastasis by using a single-head equipment (GE SPECT Millennium MPR) with a parallel-beam low-energy high-resolution (LEHR) collimator (energy peak = 140 keV, intrinsic energy resolution ≤9.5%, energy window = 20%, and intrinsic spatial resolution ≤6.9 mm). The SPECT imaging was taken between 2 and 3 h after the intravenous injection of ^99m^Tc-MDP (20–25 mCi). The imaging size was 256 × 1024 with a pixel size of 2.26 mm. The acquisition time was 10–15 min for each whole-body bone scan image.

We selected 112 images that contained bone metastasis lesions in the thorax from all images. The selected images were cropped to extract the regional images using the image cropping technique as mentioned in Section 2.1. We organized these 112 regional images into the data set D1, which is outlined in Table 1.

**TABLE 1:** Overview of the data sets used in this work.

Data set	Number of samples	Annotation
D1	112	—
D2	2280	—
D3	2280	—
D4	4560	D2 + D3

The subsequent section details the process of augmenting the size of D1, which would be helpful for training a better segmentation model since deep learning–based models often perform well on the “big” data set.

### 3.2 Data augmentation

#### 3.2.1 Geometric transformation

Geometric transformations such as flipping, cropping, rotation, and translation are frequently used in the field of deep learning–based image augmentation (Shorten, Khoshgoftaar). In this work, image flipping, rotation, and translation were applied on the images in data set D1 to obtain more samples.

The augmented samples that were obtained by using the aforementioned geometric transformations and the images in data set D1 were grouped into data set D2, which is outlined in Table 1.

#### 3.2.2 Adversarial learning

The generative adversarial network (GAN) (Goodfellow et al., 2014) is one of the most emerging deep learning techniques that is used to generate new “fake” samples with the given images. The generated samples have an entirely different distribution from the original ones. The GAN consists of a generator (*G*) and discriminator (*D*). Specifically, generator *G* generates “fake” data *G*(*z*) from a distribution of *P*_*Z*_ and discriminator *D* distinguishes fake data from real data *X*.

The deep convolutional GAN (DCGAN) (Radford et al., 2015) as a variant of GAN has the potential to improve the stability of training and alleviate mode collapse that the original GAN may suffer from. Assume that the distribution of real data is *P*_*D*_, both the generator and discriminator are iteratively optimized against each other in a minimax game as follows (Radford et al., 2015):

$$\max_{\theta_{G}}\max_{\theta_{D}}E_{x∼P_{D}}\left[\log D\left(x\right)\right]+E_{z∼p_{z}}\left[\log\left(1-D\left(G\left(z\right)\right)\right)\right],$$ (9)

where *θ*_*G*_ and *θ*_*D*_ denote the parameters of *G* and *D*, respectively.

**FIGURE 6:**
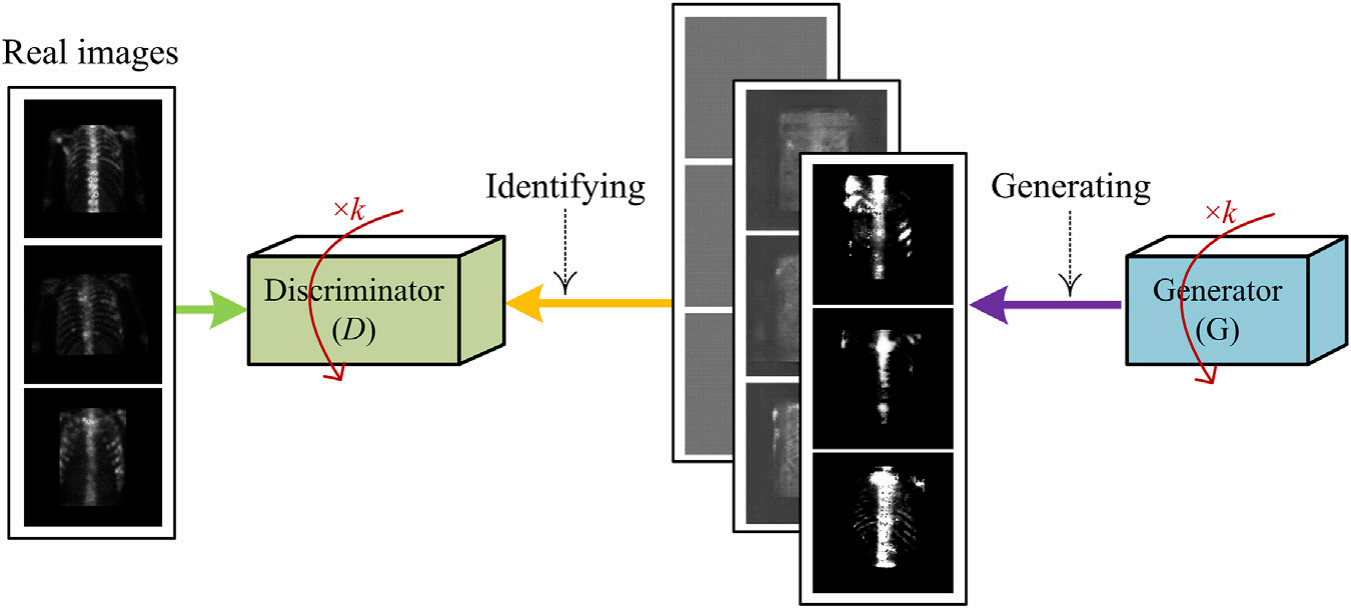
Illustration of generating fake samples of SPECT images using DCGAN-based sample generation technique.

In this work, we used a DCGAN-based technique to generate the simulated samples of SPECT images. Figure 6 shows the diagram of training such a network, where the iteration parameter is set as *k* = 3 in the experiment.

The samples generated by using the aforementioned DCGAN-based technique and the images in data set D1 are grouped into data set D3, which is outlined in Table 1.

### 3.3 Experimental setup

The evaluation metrics used include Dice similarity coefficient (*DSC*), class pixel accuracy (*CPA*), and *Recall*, which are defined in Eqs 10–12.

$$DSC=\frac{2\cdot TP}{FP+2\cdot TP+FN},$$ (10)

$$CPA=\frac{TP}{TP+FP},$$ (11)

$$Recall=\frac{TP}{TP+FN},$$ (12)

where *TP* = true positive, *TN* = true negative, *FP* = false negative, and *FN* = false negative.

The parameter setting of the proposed deep segmentation model is provided in Table 2.

**TABLE 2:** Parameter setting of the proposed deep segmentation model.

Parameter	Value
Input size	256 × 256
Optimizer	Adam
Learning rate	0.0005
Learning momentum	0.9
Weight decay	0.0001
Epoch	400

In the experiment, we divided each data set in Table 1 into two parts: subset 1# for unsupervised learning and subset 2# for supervised learning. Specifically, in each subset, we have randomly chosen 70% of the samples to train and the rest (30%) to test the developed segmentation model. It is worth noting that samples without manual labels are used to train the proposed model in an unsupervised manner, and samples with a varied number of manual labels are used to train the model in a semi-supervised manner. Images which include the augmented ones from the same patient were not divided into different subsets because they would show similarities. The trained model was run 10 times on the test subset to reduce the effects of randomness. For the aforementioned defined evaluation metrics, the final outputs of the model are the average of the 10 running results. The experimental results reported in the next section are the averaged ones, unless otherwise specified.

The experiments are run in TensorFlow 2.0 on an Inter Xeon(R) Silver 4110 PC with 16 Kernels 62 GB RAM running on Ubuntu 16.04 equipped with a GeForce RTX 2080 × 2.

### 3.4 Experimental results

In this subsection, we evaluated the segmentation performance of the proposed semi-supervised model with respect to the evaluation metrics on several data sets as shown in Table 1.

#### 3.4.1 Quantitative scores

Table 3 reports the scores of evaluation metrics obtained by the semi-supervised model on test samples in data sets D1–D4, where *L*_*label*_ refers to the number of labeled samples used while training the model.

**TABLE 3:** Scores of evaluation metrics obtained by the proposed semi-supervised model.

Data set	*L*_*label*_	DSC	CPA	Recall
D1	10	0.582	0.618	**0.547**
D2	210	**0.586**	**0.621**	0.539
D3	210	0.481	0.507	0.471
D4	210	0.483	0.514	0.487

The bold value in each column indicates the maximal one.

On the whole, the proposed semi-supervised model performs best on data set D2. This tells us, on the one hand, that data augmentation positively contributes to improving segmentation performance; on the other hand, geometric transformation is more suitable to be used for augmenting the size of the SPECT data set when compared to the adversarial learning–based augmentation since image flipping, translation, and rotation operations can preserve label post-transformation.

**FIGURE 7:**
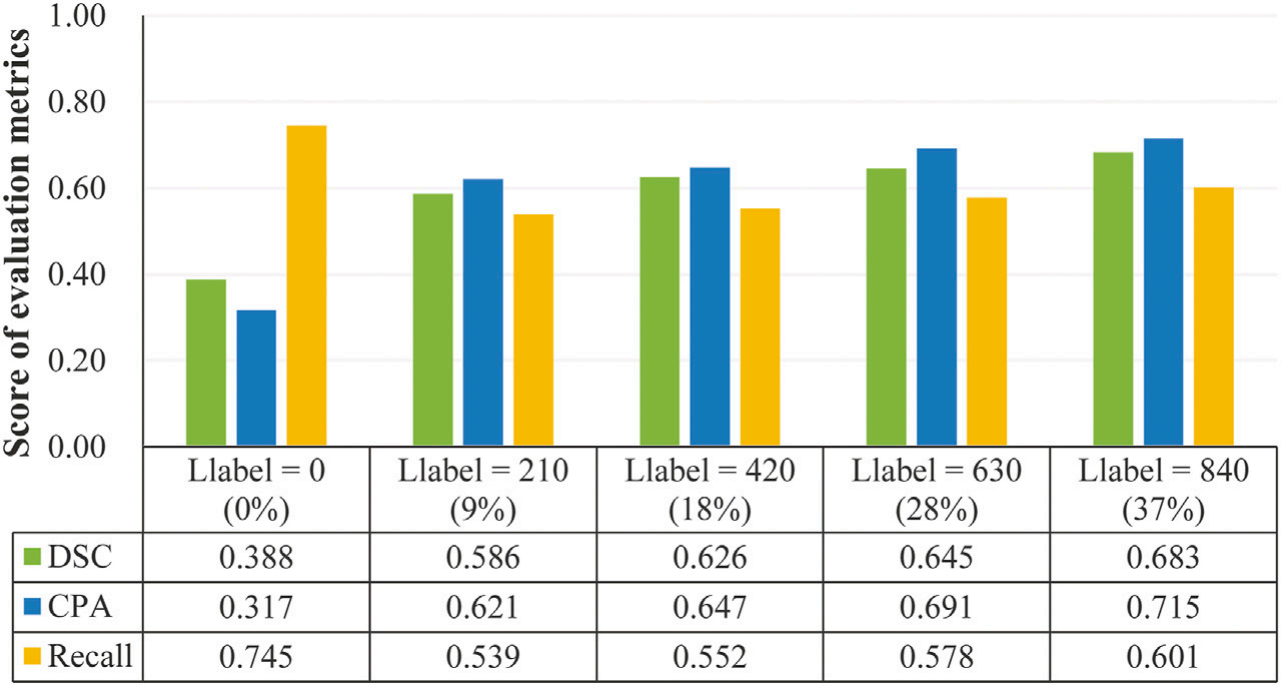
Segmentation performance obtained by the proposed model when varying numbers of labeled samples were used during training the model.

Using test samples in data set D2, we examined how the number of labeled samples (i.e., *L*_*label*_) affects the segmentation performance, by providing experimental results in Figure 7.

From the experimental results in Figure 7, we can see that as expected, the scores of evaluation metrics keep increasing as *L*_*label*_ increases. When 37% of the labeled samples were used for the training model, the best segmentation performance (*DSC* = 0.683, *CPA* = 0.715, and *Recall* = 0.601) was obtained. An exception is that the unsupervised model (*L*_*label*_ = 0) obtained the highest score for the *Recall* metric, which is mainly contributed by background pixels during testing the model. This reveals the major difference between our SPECT and the natural images: objects (i.e., lesions) in the former are far smaller than those in the background.

#### 3.4.2 Ablation experiments

The reported aforementioned experimental results were obtained when the complete model was recurrently run thrice on data set D2 without image aggregation. In this subsection, a set of scores of ablation experiments are reported.

**FIGURE 8:**
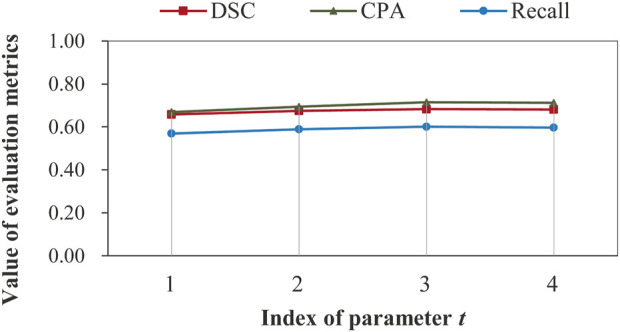
Illustration of impact of recurrent feature extraction on segmentation performance obtained by the complete model on data set D2 without image aggregation.

Impact of recurrent feature extraction on segmentation performance: as mentioned in subsection 2.4.1, the feature extraction network can run in a recurrent manner. It is necessary to examine the impact of parameter *t* on the segmentation performance, which is illustrated in Figure 8.

Figure 8 demonstrates that recurrent feature extraction is of great necessity, and a value of 3 for *t* is optimal when the complete model is used during the feature extraction stage. In Table 4, we further present the number of model parameters and the test time for different recurrence times.

**TABLE 4:** Number of model parameters and test time for different recurrence times.

*t*	Number of model parameters (million)	Test time (sec)
1	3.654	2.19
2	9.808	2.45
3	23.545	2.60
4	46.826	4.21

It can be seen that the proposed model can segment bone metastatic lesions efficiently with a maximum test time of 4.21 s. Despite the significant increases of parameters, the time grows slowly.

Impact of network structure on segmentation performance: until now, the best aforementioned segmentation performance was obtained when the feature extraction network was structured as stacked dilated residual convolutions combined with the inception block. We call such a model a *complete model*. It is required to explore whether the segmentation performance varies as the structure of the feature extraction network changes. Table 5 reports the scores of *DSC* under different cases, with each indicating one type of setting, where Case 4# denotes the one with which our model achieved the best segmentation performance.

**TABLE 5:** Impacts of structure changes of the feature extraction network on the segmentation performance.

Case	Dilated conv.	Inception	Residual unit	DSC
1#	—	—	—	0.632
2#	√	—	—	0.656
3#	√	√	—	0.667
4#	√	√	√	**0.683**

The bold value in each column indicates the maximal one.

We can see from Table 5 that the value of *DSC* increases steadily as the feature extraction network approaches the “perfect” structure as shown by Case 4#. An absolute increase of 0.051 for *DSC* shows superiority of stacked dilated residual convolution layers for automatically extracting the representative features of bone metastasis lesions from low-resolution SPECT images.

**FIGURE 9:**
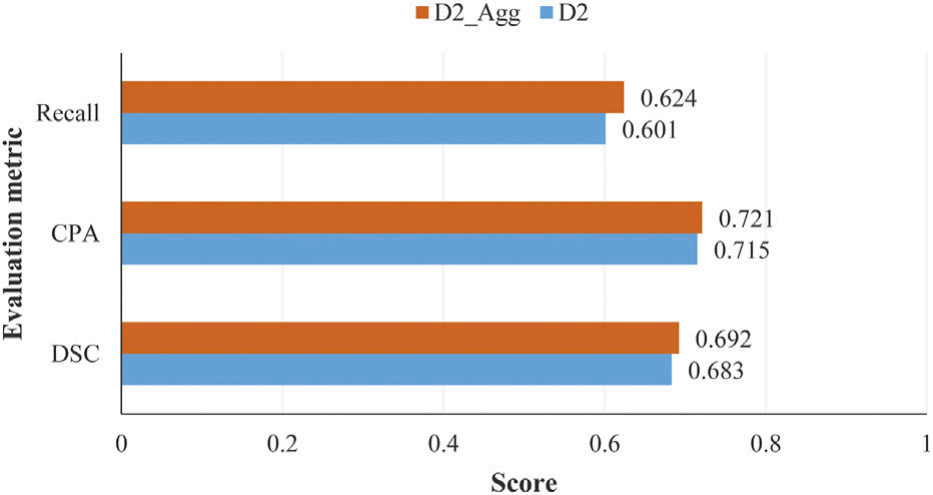
Scores of evaluation metrics obtained by the proposed model on test samples in data sets D2 and D2_Agg, with the number of labeled samples used for training the model being *L*_*label*_ = 840 and *L*_*label*_ = 420 for D2 and D2_Agg, respectively.

Impact of image aggregation on segmentation performance: image aggregation operation conducted on ‘optimal’ data set D2 outputs an aggregated data set D2_Agg. There are 1,140 aggregated samples in data set D2_Agg. Image or feature aggregation is detailed in subsection 2.1.3. In Figure 9, we have shown the scores of several evaluation metrics on data sets D2 and D2_Agg.

We can see from Figure 9 that the image aggregation operation slightly improves the performance, with an increase of 0.006 for *CPA* when the proposed model was trained in a semi-supervised manner with 840 labeled samples.

#### 3.4.3 Comparative analysis

A comparative analysis was conducted between the proposed model and existing classical models which included U-Net (Ronneberger et al., 2015) and its variant nnU-Net (Isensee et al., 2021). The U-Net and its variants are well-known supervised models. We therefore selected 70% of the samples (i.e., *L*_*label*_ = 798) for training U-Net and nnU-Net. Our semi-supervised model was trained using 37% of samples (i.e., *L*_*label*_ = 420). Table 6 reports the scores of evaluation metrics achieved by several models.

**TABLE 6:** Comparative analysis between the proposed model and existing models with test samples in data set D2_Agg.

*L*_*label*_	Model	DSC	CPA	Recall
798	U-Net	0.695	0.722	0.608
798	nnU-Net	0.757	0.772	0.712
420	Proposed	0.692	0.721	0.624

It can be seen that the existing supervised models perform slightly better than the proposed semi-supervised model. On the whole, however, our model obtains comparable performance with *DSC* = 0.692. The model U-Net and its variants were originally designed for supervised learning, which cannot be trained in a semi- or unsupervised manner. The encoder–decoder structure combined with the skip connection that the U-Net has greatly inspires us to develop better semi-supervised models using more samples of SPECT images in the near future.

## 4 Discussion

In this section, we provide a brief discussion about the proposed semi-supervised segmentation model on identifying and delineating bone metastasis lesions in ^99m^TC-MDP SPECT images. This section begins with a visual presentation of the segmented images, which is followed by an analysis on the reasons that account for the imperfect performance.

**FIGURE 10:**
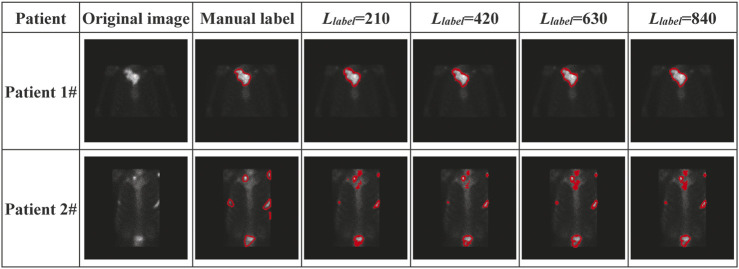
Visual presentation of manually labeled lesions and automatically segmented lesions by our semi-supervised deep segmentation model.

With regional images in the thorax acquired from two patients with metastasis, Figure 10 shows the segmented areas by our model, where the original images and manual labels are also presented.

The visual presentation depicted in Figure 10 shows that our model performs better on segmenting the single-lesion metastasis (patient 1#) than the multi-lesion one (Patient 2#). There is almost no difference between the manually labeled and the automatically delineated regions of the image acquired from Patient 1#, who was clinically diagnosed with bone metastasis in the collarbone. There is also no noticeable improvement in performance as the number of labeled samples significantly increases during the training model. By contrast, there is quite a distinction in terms of the size of the lesion between the manually labeled and automatically delineated areas of the image acquired from Patient 2#, who was clinically diagnosed with bone metastasis in the collarbone, left scapula, ribs, and lumbar vertebrae simultaneously.

Now, we present the possible reasons that negatively affect segmentation performance as follows.

Imperfect manual annotation: ^99m^TC-MDP SPECT imaging is typically characterized by the inferior spatial resolution, which brings a significant challenge to the pixel-level annotation by human researchers. The situation will get much worse when multiple tiny lesions are present in an image (e.g., the one from Patient 2# in Figure 10). Any error of manual annotation may result in incorrect classification of pixels by the automated segmentation model. The more the pixels have been correctly classified, the higher the values for *DSC*, *CPA*, and *Recall* are. Therefore, the imperfect manual annotation mainly accounts for the decreased segmentation performance.

Insufficient feature representation: the proposed deep segmentation model segments metastatic lesions *via* first extracting the hierarchical features of lesions and then classifying the pixel-level features into classes. However, learning the representative features of metastasis lesions from a small-scale data set of low-resolution images is significantly challenging as metastasis lesions are commonly distributed irregularly and typically show variability in size, shape, and intensity of radiopharmaceutical uptake. Although data augmentation, especially the geometric transformation-based operations, positively contributes to improving segmentation performance, more samples are still needed to be used for extracting deeper features of bone metastasis lesions.

Now, we can summarize that the proposed semi-supervised segmentation model has the potential to be used to automatically detect and delineate bone metastasis lesions with low-resolution SPECT images. A score of 0.683 for DSC has been obtained by the proposed deep model on the augmented data set if only 37% (≈840/2280) of the labeled samples were used for training the model. In the case that the model was trained with the aggregated samples, i.e., 37% (≈420/1140) of the labeled images, a score of 0.692 was obtained for *DSC*, achieving comparable segmentation performance.

## 5 Conclusion

To facilitate the clinical diagnosis of skeletal metastasis by nuclear medicine physicians, in this work, we have proposed a semi-supervised segmentation model to automatically detect and delineate bone metastasis lesions in the regional SPECT images. The proposed model was presented by detailing the structures of feature extraction and pixel-level feature classification stages. Experimental data of clinical SPECT bone scan images and the data augmentation methods used were also elaborated. The experimental evaluation conducted on these images has shown that the proposed model has the potential to be used as a clinical tool for automatically delineating the boundaries of bone metastasis lesions in low-resolution images, achieving a best mean score of 0.692 for *DSC*, if the model was trained using 37% of the aggregated samples with manual labels.

We plan to extend our work in two directions in the future. First, we intend to collect more data of clinical SPECT images and fine-tune the proposed semi-supervised model such that it can work in computer-aided diagnosis systems. Second, we plan to develop models for whole-body SPECT image segmentation, enabling automated detection and delineation of multi-lesion bone metastasis.

## Data Availability

The original contributions presented in the study are included in the article/Supplementary Material; further inquiries can be directed to the corresponding authors.

## References

[B1] Apiparakoon T. Rakratchatakul N. Chantadisai M. Vutrapongwatana U. Kingpetch K. Sirisalipoch S. (2020). MaligNet: Semisupervised learning for bone lesion instance segmentation using bone scintigraphy. IEEE Access 8, 27047–27066. 10.1109/access.2020.2971391

[B2] Asgari Taghanaki S. Abhishek K. Cohen J. P. Cohen-Adad J. Hamarneh G. (2021). Deep semantic segmentation of natural and medical images: A review. Artif. Intell. Rev. 54, 137–178. 10.1007/s10462-020-09854-1

[B3] Aslanta A. Dandl E. Akrolu M. Cakiroǧlu M. (2016). Cadboss: A computer-aided diagnosis system for whole-body bone scintigraphy scans. J. Cancer Res. Ther. 12 (2), 787–792. 10.4103/0973-1482.150422 27461652

[B4] Bochkovskiy A. Wang C. Y. Liao H. (2020). YOLOv4: Optimal speed and accuracy of object detection.

[B5] Chan T. F. Vese L. A. (2001). Active contours without edges. IEEE Trans. Image Process. 10 (2), 266–277. 10.1109/83.902291 18249617

[B6] Cheimariotis G. Al-Mashat M. Haris K. Aletras A. H. Jogi J. Bajc M. (2018). Automatic lung segmentation in functional SPECT images using active shape models trained on reference lung shapes from CT. Ann. Nucl. Med. 32, 94–104. 10.1007/s12149-017-1223-y 29236220 PMC5797204

[B7] Chen J. Frey E. C. (2020). Medical image segmentation via unsupervised convolutional neural network.

[B8] Cheng D. C. Hsieh T. C. Yen K. Y. Kao C. H. (2021). Lesion-based bone metastasis detection in chest bone scintigraphy images of prostate cancer patients using pre-train, negative mining, and deep learning. Diagnostics 11 (3), 518. 10.3390/diagnostics11030518 33803921 PMC8000593

[B9] Cheng D. C. Liu C. C. Hsieh T. C. Yen K. Y. Kao C. H. (2021). Bone metastasis detection in the chest and pelvis from a whole-body bone scan using deep learning and a small dataset. Electronics 10, 1201. 10.3390/electronics10101201

[B10] Christoph B. Albarqouni S. Navab N. (2017). “Semi-supervised deep learning for fully convolutional networks,” in International Conference on Medical Image Computing and Computer-Assisted Intervention, 311–319.

[B11] Costelloe C. M. Rohren E. M. Madewell J. E. Hamaoka T. Theriault R. L. Yu T. K. (2009). Imaging bone metastases in breast cancer: Techniques and recommendations for diagnosis. Lancet. Oncol. 10 (6), 606–614. 10.1016/S1470-2045(09)70088-9 19482249

[B12] Dang J. (2016). Classification in bone scintigraphy images using convolutional neural networks. master’s thesis: Lund University.

[B13] Doulamis N. Doulamis A. (2014). “Semi-supervised deep learning for object tracking and classification,” in 2014 IEEE International Conference on Image Processing (ICIP), Paris, France, 27-30 October 2014, 848–852.

[B14] Elfarra F. G. Calin M. A. Parasca S. V. (2019). Computer-aided detection of bone metastasis in bone scintigraphy images using parallelepiped classification method. Ann. Nucl. Med. 33 (11), 866–874. 10.1007/s12149-019-01399-w 31493203

[B15] Goodfellow I. J. Pouget-Abadie J. Mirza M. Xu B. Warde-Farley D. Ozair S. (2014). Generative adversarial networks. Adv. Neural Inf. Process. Syst. 3, 2672–2680.

[B16] Guo Y. Lin Q. Zhao S. Li T. Cao Y. Man Z. (2022). Automated detection of lung cancer-caused metastasis by classifying scintigraphic images using convolutional neural network with residual connection and hybrid attention mechanism. Insights Imaging 13, 24. 10.1186/s13244-022-01162-2 35138479 PMC8828823

[B17] He K. Gkioxari G. Dollar P. Girshick R. (2020). Mask R-CNN. IEEE Trans. Pattern Anal. Mach. Intell. 42 (2), 386–397. 10.1109/TPAMI.2018.2844175 29994331

[B18] Isensee F. Jaeger P. F. Kohl S. A. A. Petersen J. Maier-Hein K. H. (2021). nnU-Net: a self-configuring method for deep learning-based biomedical image segmentation. Nat. Methods 18, 203–211. 10.1038/s41592-020-01008-z 33288961

[B19] Lei T. Wang R. Wan Y. Du X. Meng H. Nandi A. (2020). Medical image segmentation using deep learning: A survey, 13120.

[B20] Li T. Lin Q. Guo Y. Zhao S. Zeng X. Man Z. (2022). Automated detection of skeletal metastasis of lung cancer with bone scans using convolutional nuclear network. Phys. Med. Biol. 67, 015004. 10.1088/1361-6560/ac4565 34933282

[B21] Liang M. Hu X. L. (2015). “Recurrent convolutional neural network for object recognition,” in 2015 IEEE Conference on Computer Vision and Pattern Recognition (CVPR), Boston, MA, 07-12 June 2015, 3367–3375.

[B22] Lin Q. Cao C. Li T. Cao Y. Man Z. Wang H. (2021). Multiclass classification of whole-body scintigraphic images using a self-defined convolutional neural network with attention modules. Med. Phys. 48 (10), 5782–5793. 10.1002/mp.15196 34455613 PMC9135133

[B23] Lin Q. Cao C. Li T. Man Z. Cao Y. Wang H. (2021). dSPIC: A deep SPECT image classification network for automated multi-disease, multi-lesion diagnosis. BMC Med. Imaging 21, 122. 10.1186/s12880-021-00653-w 34380441 PMC8359584

[B24] Lin Q. Li T. Cao C. Cao Y. Man Z. Wang H. (2021). Deep learning based automated diagnosis of bone metastases with SPECT thoracic bone images. Sci. Rep. 11, 4223. 10.1038/s41598-021-83083-6 33608560 PMC7896065

[B25] Lin Q. Luo M. Gao R. Li T. Man Z. Cao Y. (2020). Deep learning based automatic segmentation of metastasis hotspots in thorax bone SPECT images. PLoS ONE 15 (12), e0243253. 10.1371/journal.pone.0243253 33270746 PMC7714246

[B26] Litjens G. Kooi T. Bejnordi B. E. Setio A. A. A. Ciompi F. Ghafoorian M. (2017). A survey on deep learning in medical image analysis. Med. Image Anal. 42, 60–88. 10.1016/j.media.2017.07.005 28778026

[B27] Mac A. Fgeb C. Svp D. (2021). Object-oriented classification approach for bone metastasis mapping from whole-body bone scintigraphy. Phys. Med. 84, 141–148. 10.1016/j.ejmp.2021.03.040 33894584

[B28] Minaee S. Boykov Y. Porikli F. Plaza A. Kehtarnavaz N. Terzopoulos D. (2022). Image segmentation using deep learning: A survey. IEEE Trans. Pattern Anal. Mach. Intell. 44 (7), 3523–3542. 10.1109/TPAMI.2021.3059968 33596172

[B29] Moon D. H. Maddahi J. Silverman D. H. Glaspy J. A. Phelps M. E. Hoh C. K. (1998). Accuracy of whole-body fluorine-18-FDG PET for the detection of recurrent or metastatic breast carcinoma. J. Nucl. Med. 39 (3), 431–435. 9529287

[B30] Nathan M. Gnanasegaran G. Adamson K. Fogelman I. (2013). Bone scintigraphy: Patterns, variants, limitations and artefacts. Berlin Heidelberg: Springer.

[B31] Papandrianos N. Papageorgiou E. Anagnostis A. (2020). Development of Convolutional Neural Networks to identify bone metastasis for prostate cancer patients in bone scintigraphy. Ann. Nucl. Med. 34, 824–832. 10.1007/s12149-020-01510-6 32839920

[B32] Papandrianos N. Papageorgiou E. Anagnostis A. Papageorgiou K. (2020). Bone metastasis classification using whole body images from prostate cancer patients based on convolutional neural networks application. PLoS ONE 15 (8), e0237213. 10.1371/journal.pone.0237213 32797099 PMC7428190

[B33] Papandrianos N. Papageorgiou E. Anagnostis A. Papageorgiou K. (2020). Efficient bone metastasis diagnosis in bone scintigraphy using a fast convolutional neural network architecture. Diagnostics 10 (8), 532. 10.3390/diagnostics10080532 PMC7459937 32751433

[B34] Pi Y. Zhao Z. Xiang Y. Li Y. Cai H. Yi Z. (2020). Automated diagnosis of bone metastasis based on multi-view bone scans using attention-augmented deep neural networks. Med. Image Anal. 65, 101784. 10.1016/j.media.2020.101784 32763793

[B35] Radford A. Metz L. Chintala S. (2015). Unsupervised representation learning with deep convolutional generative adversarial networks.

[B36] Redmon J. Farhadi A. (2018). YOLOv3: An incremental improvement.

[B37] Ronneberger O. Fischer P. Brox T. (2015). “U-Net: Convolutional networks for biomedical image segmentation,” in International Conference on Medical Image Computing and Computer-Assisted Intervention, 234–241.

[B38] Sadik M. Hamadeh I. Nordblom P. Suurkula M. Hoglund P. Ohlsson M. (2008). Computer–assisted interpretation of planar whole-body bone scans. J. Nucl. Med. 49, 1958–1965. 10.2967/jnumed.108.055061 18997038

[B39] Sadik M. Jakobsson D. Olofsson F. Ohlsson M. Suurkula M. Edenbrandt L. (2006). A new computer-based decision-support system for the interpretation of bone scans. Nucl. Med. Commun. 27, 417–423. 10.1097/00006231-200605000-00002 16609352

[B40] Sderlund V. (1996). Radiological diagnosis of skeletal metastases. Eur. Radiol. 6, 587–595. 10.1007/BF00187654 8934120

[B41] Shan H. Jia X. Yan P. Li Y. Paganetti H. Wang G. (2020). Synergizing medical imaging and radiotherapy with deep learning. Mach. Learn, Sci. Technol. 1, 021001. 10.1088/2632-2153/ab869f

[B42] Shorten C. Khoshgoftaar T. M. (2019). A survey on image data augmentation for deep learning. J. Big Data 6, 60. 10.1186/s40537-019-0197-0 PMC8287113 34306963

[B43] Szegedy C. Liu W. Jia Y. Sermanet P. Reed S. Anguelov D. (2014). “Going deeper with convolutions,” in 2015 IEEE Conference on Computer Vision and Pattern Recognition (CVPR).

[B44] Tarvainen A. Valpola H. (2017). “Mean teachers are better role models: Weight-averaged consistency targets improve semi-supervised deep learning results,” in Proceedings of the 31st International Conference on Neural Information Processing Systems, 1–10.

[B45] Thorwarth R. Dewaraja Y. Wilderman S. Kaminski M. Avram A. Roberson P. (2013). SU-E-J-186: Automated SPECT based segmentation for quality assurance of CT-delineated tumor volumes for 131I tositumomab therapy of non-hodgkins lymphoma. Med. Phys. 40, 194. 10.1118/1.4814398

[B46] Yu F. Koltun V. (2015). “Multi-scale context aggregation by dilated convolutions,” in International Conference on Learning Representations.

[B47] Zhao Z. Pi Y. Jiang L. S. Xiang Y. Wei J. Yang P. (2020). Deep neural network based artificial intelligence assisted diagnosis of bone scintigraphy for cancer bone metastasis. Sci. Rep. 10, 17046. 10.1038/s41598-020-74135-4 33046779 PMC7550561

[B48] Zhu C. Tian L. Chen P. Wang L. Ye G. Mao Z. (2008). Application of GVF snake model in segmentation of whole body bone SPECT image. J. Biomed. Eng. 25 (1), 27–29. 18435250

